# Supplementary material for: Tumor Angiogenic Inhibition Triggered Necrosis (TAITN) in Oral Cancer
Source: Cells. 2019 Jul 22;8(7):761. doi: 10.3390/cells8070761 (PMC6678844; doi:10.3390/cells8070761)
Supplement: Supplementary file 1 [file cells-08-00761-s001.pdf]

# Supplementary Materials: Tumor Angiogenic Inhibition Triggered Necrosis (TAITN) in Oral Cancer

**Saori Yoshida, Hotaka Kawai, Takanori Eguchi, May Wathone Oo, Chang Anqi, Kiyofumi Takabatake, Shintaro Sukegawa, Keisuke Nakano, Kuniaki Okamoto, Hitoshi Nagatsuka**

**Table S1.** Stage classification.

| Stage 0 | Tis | N0 | M0 |
|---|---|---|---|
| Stage I | T1 | N0 | M0 |
| Stage II | T2 | N0 | M0 |
| Stage III | T3 | N0 | M0 |
| | T1, T2, T3 | N1 | M0 |
| Stage IV a | T4a | N0, N1 | M0 |
| | T1, T2, T3, T4a | N2 | M0 |
| Stage IV b | Any T | N3 | M0 |
| | T4b | Any N | M0 |
| Stage IV c | Any T | Any N | M1 |

Assessment of the primary tumor (T), assessment of the regional lymph nodes (N), assessment of distant metastasis (M), and Stage criteria follow the TNM classification defined in Malignant Tumors 8th Edition, published by Union International Cancer Control [30].

**Table S2.** Pathological classification of Tumor (pT classification).

| Class | Criteria |
|---|---|
| pTis | Carcinoma in situ |
| pT0 | No evidence of primary tumour |
| pT1 | Tumour 2 cm or less in greatest dimension and 5 mm or less depth of invasion |
| pT2 | Tumour 2 cm or less in greatest dimension and more than 5 mm depth of invasion or tumour more than 2 cm but not more than 4 cm in greatest dimension and depth of invasion no more than 10 mm |
| pT3 | Tumour more than 2 cm but not more than 4 cm in greatest dimension and depth of invasion more than 10 mm or tumour more than 4 cm in greatest dimension and not more than 10 mm depth of invasion |
| pT4 (oral cavity) | Tumour more than 4 cm in greatest dimension and more than 10 mm depth of invasion or tumour invades through the cortical bone of the mandible or maxilla or involves the maxillary sinus, or invades the skin of the face. |

**Table S3.** Pathological classification of regional lymph node (pN classification).

| Class | Criteria |
|---|---|
| pN1 | Metastasis in a single ipsilateral lymph node, 3 cm or less in greatest dimension without extranodal extension |

| | |
|---|---|
| pN2a | Metastasis in a single ipsilateral lymph node, 3cm or less in greatest dimension with extranodal extension or more than 3 cm but not more than 6 cm in greatest dimension without extranodal extension |
| pN2b | Metastasis in multiple ipsilateral lymph nodes, none more than 6cm in greatest dimension without extranodal extension |
| pN2c | Metastasis in bilateral or contralateral lymph nodes, none more than 6cm in greatest dimension without extranodal extension |
| pN3a | Metastasis in a lymph node more than 6cm in greatest dimension without extranodal extension |
| pN3b | Metastasis in a lymph node more than 3cm in greatest dimension with extranodal extension or, multiple ipsilateral, or any contralateral or bilateral node with extranodal extension |
